# Concordance of renal tumour assessments by urologists using CT scans versus Hyper‐Accuracy 3D Virtual Models for surgical planning: A single‐centre multireviewer analysis

**DOI:** 10.1002/bco2.70002

**Published:** 2025-05-07

**Authors:** Francesco Ditonno, Michele Boldini, Francesco Cianflone, Lorenzo Treccani, Lorenzo De Bon, Francesca Fumanelli, Francesco Artoni, Claudio Brancelli, Iolanda Palumbo, Alberto Baielli, Alberto Bianchi, Filippo Migliorini, Riccardo Bertolo, Alessandro Veccia, Alessandro Antonelli

**Affiliations:** ^1^ Department of Urology University of Verona, Azienda Ospedaliera Universitaria Integrata Verona Italy

**Keywords:** anatomical models, partial nephrectomy, renal cancer, robotic surgical procedures, surgical planning, virtual reality

Robot‐assisted partial nephrectomy (RAPN) might represent a surgically demanding procedure that conceals several pitfalls, including proximity to vessels or calyces and lesions not visible upon kidney exposure. Therefore, a comprehensive understanding of key anatomical landmarks is crucial for precise surgical planning and procedural success. Several innovative technological tools have been proposed, including three‐dimensional virtual models (3DVMs).^1^ Their routine use could enhance preoperative and intraoperative guidance, broadening the indication for RAPN.^2^

The primary aim of the present study was to evaluate the inter‐rater agreement of urologists interpreting conventional CT scans versus hyperaccuracy (HA)‐3DVMs (MEDICS Srl, Turin, Italy) to guide preoperative planning for renal masses.

A prospectively maintained database of patients undergoing kidney surgery for renal masses at our Institution was queried to retrieve data of all consecutive RAPN interventions, with preoperative CT scans and HA‐3DVMs available. Patients with a history of prior renal surgery and bilateral or multiple ipsilateral tumours were discarded. Performance of CT scans followed a standard internal protocol for staging solid renal masses, using a contrast‐medium multidetector CT with 3/5‐mm sections from the pulmonary base to the pelvis, with basal, arterial, venous, and excretory phases. The respective HA‐3DVMs were developed in selected cases as previously described,^3^ based on the expected surgical complexity.

The primary outcome was interobserver agreement across 12 specific preoperative surgical planning domains, assessed using a custom‐designed questionnaire (Supplementary Material).^4^ The questionnaire comprised 12 items covering different aspects of preoperative surgical planning. Twelve urologists, including six residents and six experienced practitioners, evaluated each clinical case using both the CT scan and the respective HA‐3DVMs.

Interobserver agreement was measured using Cohen's kappa (k) statistics for each domain across multiple raters, with 95% confidence intervals (CI) determined by 1000 bootstrap repetitions. Kappa values could range from 0 to 1, with agreement defined as almost perfect (*k* > 0.8), substantial (*k* > 0.6), moderate (*k* > 0.4), fair (*k* > 0.2), or none to slight (*k* < 0.2).^5^ Each clinician evaluated all 31 cases twice: once using CT scans and once with HA‐3DVMs. The order of evaluation (CT first or HA‐3DVM first) was left to clinicians' preference. Each imaging modality was reviewed independently, without back‐to‐back comparisons, to ensure an unbiased assessment. The time required to complete the evaluation of either the CT scan or HA‐3DVMs was recorded. Differences in evaluation times were estimated using the Wilcoxon signed‐rank test. A subgroup analysis was conducted to evaluate the interobserver agreement separately for attendings and residents, stratified by imaging modality, to identify potential differences in agreement patterns between these subgroups. The Stata® 17.0 software (StataCorp LLC, College Station, TX, USA) was used for statistical analysis with statistical significance set at *p* < 0.05.

A HA‐3DVM was performed for 31 patients. The median tumour size was 6 cm (IQR 3.5–7.2). RAPN was successfully performed in 25 patients (80.6%). In 6 patients, radical nephrectomy was performed instead. Cohen's kappa coefficient showed higher agreement for HA‐3DVMs across most questions, except for those concerning the involvement of the collecting system (Q7, kappa coefficient: 0.37 vs. 0.36) and potential intraoperative opening of the excretory structures (Q12, kappa coefficient: 0.30 vs. 0.26), where the coefficients were nearly identical. Interobserver agreement based on preoperative CT scans was moderate for items concerning tumour position (Q1–3), exophytic/endophytic properties (Q4) and clamping strategy (Q10), and fair or lower for the remaining questions. In contrast, interobserver agreement for HA‐3DVMs was substantial for the tumour's longitudinal position (Q1) and its exophytic/endophytic properties (Q4). Moderate agreement was observed for the antero‐posterior (Q2) and lateral (Q3) positions, as well as for the necessity and duration of vascular clamping (Q10), while a fair agreement was found for the rest of the questions (Q5–9, Q11–14) (Figure 1). When comparing attendings and residents, interobserver agreement was substantial for HA‐3DVM interpretation (attendings: *κ* = 0.69; residents: *κ* = 0.67), irrespective of physician status, compared to a moderate to fair agreement for CT scan evaluation (attendings: *κ* = 0.41; residents: *κ* = 0.29). Furthermore, nearly all examiners (10/12, 83.3%) required significantly less time to review HA‐3DVMs than CT scan imaging.

**FIGURE 1:**
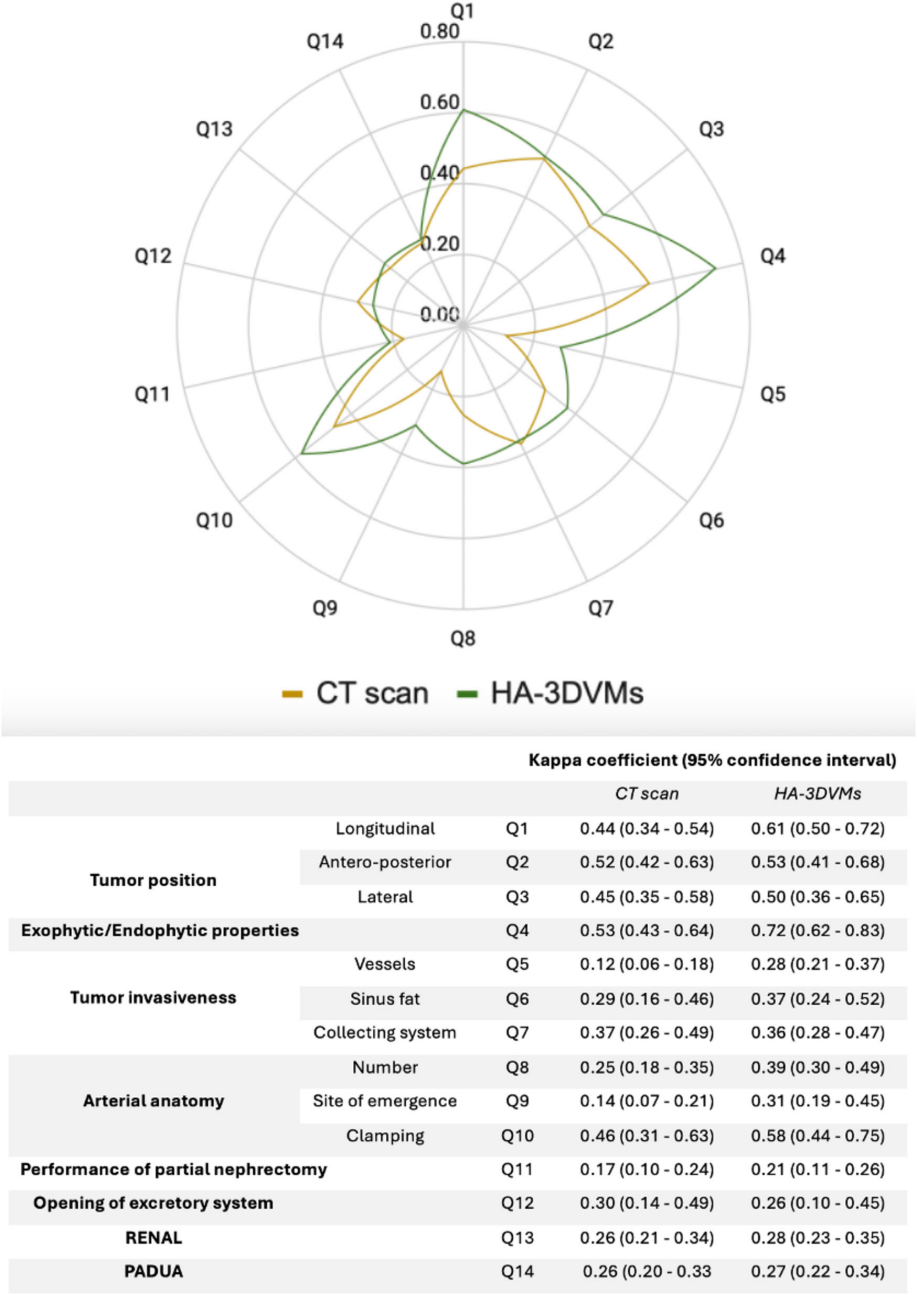
Interobserver variability of CT scan compared to hyperaccuracy 3D virtual model (HA‐3DVMs).

According to our findings, HA‐3DVMs showed higher interobserver agreement than CT scans and shorter evaluation times. To our knowledge, this study represents one of the few attempts to compare the interobserver agreement between CT scans and HA‐3DVMs. These results provide additional evidence supporting the role of HA‐3DVMs as a facilitator of surgical planning.

This virtual technology facilitates the understanding of anatomical details by eliminating the mental translation of two‐dimensional cross‐sectional imaging into three‐dimensional spatial structures of the kidney. Consequently, it overcomes the loss of spatial depth perception associated with two‐dimensional imaging, enabling direct interaction between the surgeon and kidney in three dimensions.^6^ In the present study, the highest interobserver agreement was recorded for tumour topography and clamping strategy, attributable to reduced subjectivity in the imaging review. Moreover, when comparing interobserver agreement by exam type between attendings and residents, it was higher for HA‐3DVMs, irrespective of physician status. This suggests that virtual models may enhance interobserver agreement, regardless of evaluators' experience. Our findings align with existing literature, according to which the primary benefits related to presurgical 3DVMs use lie in the in‐depth understanding of the renal mass position, with an improvement in the quality of the resection and an increased adoption of a selective clamping strategy.^1^, ^2^ Through a realistic perception of three‐dimensionality, virtual models increase engagement between the examiner and the displayed image. Moreover, the possibility of breaking the model down into its essential components enables precise reconstruction of the spatial relationships between renal mass and anatomy.

Another point worth discussing is the evaluation time, which was significantly shorter for HA‐3DVMs for more than 80% of the examiners. These differences highlight that HA‐3DVMs offer a more efficient understanding of the anatomy “at a glance” than interpreting multiple plain images. Indeed, according to our results, trainees had evaluation times that closely resembled those of more experienced urologists, suggesting improved navigation of the images, despite lesser surgical experience. This enhanced efficiency can improve confidence in the surgical approach among less experienced surgeons, with great utility in the training setting.^7^ Given the substantial learning curve related to RAPN,^8^ implementing HA‐3DVMs can make teaching efforts more effective, expedite learning, and overcome challenges related to robotic training, such as competing resources and increased costs.^9^, ^10^

Furthermore, the technical complexity of RAPN for endophytic masses can explain the increased renal damage compared to ablative approaches.^11^ Several intraoperative factors, such as vascular clamping^12^ and the tension created by renorrhaphy^13^ on healthy parenchyma, are under the spotlight for their contribution to additional tissue loss. Consequently, ongoing interest is in modifying this technique to minimize damage to healthy tissue. In this context, adding HA‐3DVMs to the preoperative work‐up can represent a valuable tool in the urologist's armamentarium, maximizing procedural efficiency and enhancing surgical and functional outcomes.

In conclusion, our study highlights the advantages of incorporating HA‐3DVMs into the preoperative assessment of renal masses. Enhanced interobserver agreement and reduced evaluation times underscore the potential of HA‐3DVMs to offer a more intuitive and accurate surgical approach, improving preoperative and intraoperative decision‐making.

## AUTHOR CONTRIBUTIONS

*Conception and design*: Francesco Ditonno, Alessandro Veccia, Alessandro Antonelli. *Acquisition of data*: Francesco Ditonno, Michele Boldini, Francesco Cianflone, Lorenzo Treccani, Lorenzo De Bon, Francesca Fumanelli, Francesco Artoni, Claudio Brancelli, Iolanda Palumbo, Alberto Baielli, Alberto Bianchi, Filippo Migliorini, Riccardo Bertolo, Alessandro Veccia, Alessandro Antonelli. *Analysis and interpretation of data*: Francesco Ditonno, Alessandro Veccia. *Drafting of the manuscript*: Francesco Ditonno. *Critical revision of the manuscript for important intellectual content*: Francesco Ditonno, Michele Boldini, Francesco Cianflone, Lorenzo Treccani, Lorenzo De Bon, Francesca Fumanelli, Francesco Artoni, Claudio Brancelli, Iolanda Palumbo, Alberto Baielli, Alberto Bianchi, Filippo Migliorini, Riccardo Bertolo, Alessandro Veccia, Alessandro Antonelli. *Supervision*: Alessandro Antonelli. *Final approval of the version to be published*: Francesco Ditonno, Michele Boldini, Francesco Cianflone, Lorenzo Treccani, Lorenzo De Bon, Francesca Fumanelli, Francesco Artoni, Claudio Brancelli, Iolanda Palumbo, Alberto Baielli, Alberto Bianchi, Filippo Migliorini, Riccardo Bertolo, Alessandro Veccia, Alessandro Antonelli. *Agreement to be accountable for all aspects of the work in ensuring that questions related to the accuracy or integrity of any part of the work are appropriately investigated and resolved*: Francesco Ditonno, Michele Boldini, Francesco Cianflone, Lorenzo Treccani, Lorenzo De Bon, Francesca Fumanelli, Francesco Artoni, Claudio Brancelli, Iolanda Palumbo, Alberto Baielli, Alberto Bianchi, Filippo Migliorini, Riccardo Bertolo, Alessandro Veccia, Alessandro Antonelli.

## CONFLICT OF INTEREST STATEMENT

The authors have no conflict of interest to declare.

## ETHICS STATEMENT

All patients provided informed consent before inclusion in the study. The study was conducted in accordance with the principles of the Helsinki Declaration.

## Supporting information


**Data S1.** Supporting Information

## Data Availability

The data sets generated during and/or analysed during the current study are available from the corresponding author on reasonable request.

## References

[1] Piramide F , Kowalewski KF , Cacciamani G , Rivero Belenchon I , Taratkin M , Carbonara U , et al. Three‐dimensional model‐assisted minimally invasive partial nephrectomy: a systematic review with meta‐analysis of comparative studies. Eur Urol Oncol. 2022;5(6):640–650. 10.1016/j.euo.2022.09.003 36216739

[2] Bertolo R , Autorino R , Fiori C , Amparore D , Checcucci E , Mottrie A , et al. Expanding the indications of robotic partial nephrectomy for highly complex renal tumors: urologists' perception of the impact of hyperaccuracy three‐dimensional reconstruction. J Laparoendosc Adv Surg Tech. 2019 Feb;29(2):233–239. 10.1089/lap.2018.0486 30394820

[3] Porpiglia F , Fiori C , Checcucci E , Amparore D , Bertolo R . Hyperaccuracy three‐dimensional reconstruction is able to maximize the efficacy of selective clamping during robot‐assisted partial nephrectomy for complex renal masses. Eur Urol. 2018;74(5):651–660. 10.1016/j.eururo.2017.12.027 29317081

[4] Antonelli A , Veccia A , Palumbo C , Peroni A , Mirabella G , Cozzoli A , et al. Holographic reconstructions for preoperative planning before partial nephrectomy: a head‐to‐head comparison with standard CT scan. Urol Int. 2019;102(2):212–217. 10.1159/000495618 30540991

[5] McHugh ML . Interrater reliability: the kappa statistic. Biochem Med Zagreb. 2012;22(3):276–282. 10.11613/BM.2012.031 23092060 PMC3900052

[6] Bertolo R , Hung A , Porpiglia F , Bove P , Schleicher M , Dasgupta P . Systematic review of augmented reality in urological interventions: the evidences of an impact on surgical outcomes are yet to come. World J Urol. 2020;38(9):2167–2176. 10.1007/s00345-019-02711-z 30826888

[7] Scott ER , Singh A , Quinn AM , Morano S , Karp A , Boyd K , et al. The use of individualized 3D‐printed models on trainee and patient education, and surgical planning for robotic partial nephrectomies. J Robot Surg. 2022;17(2):465–472. 10.1007/s11701-022-01441-6 35781195

[8] Larcher A , Muttin F , Peyronnet B , De Naeyer G , Khene ZE , Dell'Oglio P , et al. The learning curve for robot‐assisted partial nephrectomy: impact of surgical experience on perioperative outcomes. Eur Urol. 2019;75(2):253–256. 10.1016/j.eururo.2018.08.042 30243798

[9] Grover S , Tan GY , Srivastava A , Leung RA , Tewari AK . Residency training program paradigms for teaching robotic surgical skills to urology residents. Curr Urol Rep. 2010;11(2):87–92. 10.1007/s11934-010-0093-9 20425095

[10] Hermans T , Snoeks JM , vom Dorp F , Wiesner C , Steiner T , von Rundstedt F . Validation of a 3d‐printed robot‐assisted partial nephrectomy training model. BJUI Compass. 2024;5(1):90–100. 10.1002/bco2.269 38179024 PMC10764170

[11] Pandolfo SD , Beksac AT , Derweesh I , Celia A , Schiavina R , Bianchi L , et al. Percutaneous ablation *vs* robot‐assisted partial nephrectomy for completely endophytic renal masses: a multicenter trifecta analysis with a minimum 3‐year follow‐up. J Endourol. 2023;37(3):279–285. 10.1089/end.2022.0478 36367175

[12] Antonelli A , Cindolo L , Sandri M , Veccia A , Annino F , Bertagna F , et al. Is off‐clamp robot‐assisted partial nephrectomy beneficial for renal function? Data from the CLOCK trial. BJU Int. 2022;129(2):217–224. 10.1111/bju.15503 34086393

[13] Bertolo R , Ditonno F , Veccia A , De Marco V , Migliorini F , Porcaro AB , et al. Single‐layer versus double‐layer renorrhaphy technique during robot‐assisted partial nephrectomy: impact on perioperative outcomes, complications, and functional outcomes. Minerva Urol Nephrol. 2024;76(2):176–184. 10.23736/S2724-6051.24.05700-8 38742552

