# Supplementary material for: Concordance of renal tumour assessments by urologists using CT scans versus Hyper‐Accuracy 3D Virtual Models for surgical planning: A single‐centre multireviewer analysis
Source: BJUI Compass. 2025 May 7;6(5):e70002. doi: 10.1002/bco2.70002 (PMC12056593; doi:10.1002/bco2.70002)
Supplement: Supplementary file 1 — Data S1. Supporting Information [file BCO2-6-e70002-s001.docx]

**Supplementary Material**

**Q1.** Longitudinal position:

- Upper pole
- Upper third
- Middle third
- Lower third
- Lower pole

**Q2.** Antero-posterior position:

- Entirely anterior
- Intermediate
- Entirely posterior

**Q3.** Lateral position:

- Medial
- Intermediate
- Lateral

**Q4.** Exophitic/endophitic properties:

- >50% exophytic
- >50% endophytic
- Entirely endophytic

**Q5.** Vascular involvement:

- Main artery
- Artery branches
- Main vein
- Vein branches
- None

**Q6.** Sinus fat involvement:

- No
- Yes

**Q7.** Proximity/Invasion of the collecting system:

- Calix
- Pelvis
- Ureter
- No

**Q8.** Arterial anatomy

- Normal
- Supernumerary
- Accessory
- Early division
- Other (specify)

**Q9.** Site of emergence with respect to the renal vein:

- Cranial
- Caudal
- Normal
- Anterior
- Posterior

**Q10.** Artery clamping:

- No
- 5-10 min
- 10-15 min
- 15-20 min
- >20min

**Q11.** Performance of partial nephrectomy

- No
- Yes

**Q12.** Opening of the excretory system:

- No
- Yes

**Q13.** RENAL score

**Q14.** PADUA score
